# How natural light influences HSR drivers’ visual behavior

**DOI:** 10.3389/fpubh.2025.1555387

**Published:** 2025-03-24

**Authors:** Pengfei Li, Tianrun Gao, Zhuodong Liu, Boyu Liu, Qian Li, Jing Luan, Qun Chen, Jianjun Zhu

**Affiliations:** ^1^The State Key Laboratory of Heavy Duty AC Drive Electric Locomotive Systems Integration, Zhuzhou, China; ^2^CRRC Zhuzhou Locomotive Co., Ltd., Zhuzhou, China; ^3^School of Economics and Management, Beijing Jiaotong University, Beijing, China

**Keywords:** natural light, illuminance, K-means clustering, HSR drivers, visual behavior, occupational safety

## Abstract

Existing studies have shown that the lighting environment is essential in influencing a driver’s visual behavior. Due to the pivotal role of high-speed railway (HSR) in worldwide transit, it is necessary to examine how HSR drivers’ visual behavior adjust under different lighting environments. However, the methods for evaluating and categorizing lighting conditions have not been fully explored. In this study, we established a general framework for examining the impact of lighting on driver’s visual behavior. The application of this framework to explore the effects of natural light on HSR drivers’ visual characteristics was elaborated. Particularly, we used unsupervised machine learning methods to classify natural light conditions automatically. Specifically, Fuxing HSR simulation, illuminance meter, and Tobii Nano eye-tracker were employed to collect data. K-means clustering analysis of daily illuminance data identified 3 natural light conditions, namely low illuminance (1 _pm_–6 _pm_), medium illuminance (6 _am_–9 _am_), by and high illuminance (9 _am_–1 _pm_). Further, ANOVA with 3 natural light environments * 2 tunnel conditions * 4 areas of interest (AOIs) were conducted. Results manifested drivers’ visual characteristics under different natural light conditions. Specifically, lower illuminance can lead to a wider average pupil diameter, while higher illuminance results in a greater number of fixations and saccades, and a shorter time to first fixation. Moreover, all the eye movement indicators are highest for the speed dial AOI. This study contributes to the field by developing a framework to examine the effects of lighting on drivers’ visual behavior. The findings provide new insights into analyzing lighting environments by using machine learning methods, which servers to HSR driving safety and operational management.

## 1 Introduction

High-speed railway (HSR) is a wide and popular public transportation around the world due to its rapidity, reliability, and eco-friendliness (1). All new lines of the HSR network in China have a designed speed of 250 km/h and some lines are over 200 km/h (2, 3), which raises a higher need for drivers’ vigilance and vision. HSR driving shares similarities with normal trains and light rail, but imposes distinct operational demands due to its elevated velocities. The increased speed not only amplifies required attention level and cognitive workload, but also intensifies environmental challenges including rapid variation of natural lighting (1). Although the HSR cabins incorporate double-curved windshield designs aimed at mitigating driver’s visual burden caused by light refraction and deformation, drivers still encounter substantial operational challenges. These include maintaining operational safety in high-pressure driving environments, remaining vigilance against dynamic hazards (avian incursions, sandstorm conditions, etc.), and ensuring high-quality services through exact station alignment and schedule adherence. These heavily rely on drivers’ visual performance.

The Federal Railway Administration (FRA) has indicated that human error accounts for approximately 38% of railway accidents, with a significant 40% of these incidents resulting from insufficient alertness (3). Visual characteristics are one of the most important indicators to reflect the changes of driver’s vigilance. Current research has demonstrated that vision is the predominant means through which drivers interpret external conditions (like natural lighting changes), and it’s playing a crucial role in ensuring driving safety (4, 5). For example, Du et al. (6) found that accidents happen frequently during the day between 8 am and noon. Increased luminance levels significantly enhanced drivers’ reaction speed, and both reaction speed and its rate of improvement show exponential growth corresponding to the elevation of luminance intensity (7). However, most of the research is about the road driver.

Extant research about the effect of lighting on drivers’ visual behavior can be grouped into two main categories. One tendency focuses on the effects of the general lighting environment on drivers’ visual behavior, which covers daytime, nighttime, and different weather (e.g., sunny, cloudy days). For example, Li et al. (8) developed a dynamic illumination method by considering traffic flow changes for rural highway intersections to reduce nighttime accidents. Law and Petric (9) adopted Bayesian spatial analysis to discuss the trends in space and time for Day-Dark KSI risk change by analyzing publicly available data on traffic collisions. The results of Bassani et al. (10) research indicated that drivers’ average speeds and deviations were significantly affected by changes in lighting parameters for the different conditions (sunny, cloudy, and dark). The other concerns on special lighting conditions, particularly tunnel lighting, including inside the tunnel, long vs. short tunnels, etc. Peng et al. (11) found that lighting in tunnels not only impacts the driver’s visual performance but also causes physiological fatigue and mental stress. Deng et al. (12) proposed a numerical-based approach to test the influence of lighting distribution inside the tunnel on the driver’s driving task. Jiao et al. (13, 14) explored the different influence between the lighting environments of extra-long undersea tunnels and ordinary highway tunnels on driver.

Existing studies about evaluating and categorizing lighting conditions usually use simple taxonomy, such as day vs. night (8, 13), sunny vs. cloudy days (12), and long vs. short tunnels (9). Upon examining the relationship between the lighting environment and driver behavior, it is crucial to employ a reliable method for evaluating and categorizing the lighting conditions. This research focuses on classifying lighting environments, utilizing an enhanced and automated technique for identifying everyday natural light conditions: the K-means clustering method. The K-means algorithm, a classic example of unsupervised machine learning, operates on the principle that similar items tend to group. It segments a collection of physical or conceptual items into distinct clusters, each containing objects with shared characteristics. This algorithm stands out as a quintessential clustering technique. Utilizing K-means clustering, this research endeavors to automatically discern the daily variations in natural lighting conditions. This analysis is pivotal in exploring the effects of natural light on the visual behavior of HSR drivers. In this context, a natural light environment refers to the light emanating from sunrise to sunset, disregarding any artificial lighting systems.

Moreover, this research underscores the visual behavior of HSR drivers due to consideration of driving safety. Speed is the most distinctive feature of HSR, significantly distinguishing them from other general trains (15). First, under such high speeds, safe driving is closely related to the HSR drivers’ dynamic visual behavior. Current research has demonstrated that safe driving hinges on the driver’s visual attention (16). Second, the high speed coupled with substantial mental and cognitive demands causes great stress on HSR drivers’ visual behavior, which is directly related to the reaction speed of hazard perception (17). Specifically, HSR drivers must pay robust attention to the track conditions and driving speed, interact with the onboard human-machine interface, and maintain communication with dispatchers. These lead to their high mental and cognitive load, which can impact their reaction to danger very much. Third, paying attention to drivers’ visual behavior is conducive to the prevention and early warning of the occurrence of major accidents.

We specifically focus on drivers’ visual characteristics. These characteristics denote the fluctuations in drivers’ visual responses to environmental changes during the driving process, which are instrumental in assessing their driving conditions (18). There are typically four key indicator categories: those pertaining to the pupil (such as pupil diameter), those related to fixations (e.g., fixation numbers), those concerning saccades (such as saccade numbers), and those associated with blinking (e.g., blink numbers). Pupil-related metrics are indicative of variations in luminance. Fixation-related measures are linked to cognitive engagement and concentration. Saccade-related metrics provide insights into an individual’s visual processing tactics. Blink-related measures are useful for assessing levels of attention and fatigue. For this study, considering research purpose and driving tasks, our primary focus is on the first three types of visual indicators. In conclusion, this study aims to establish a methodological framework for exploring the impact of lighting on drivers’ visual behavior. Particularly, we introduce a practical and accessible approach for automatically categorizing natural light environments. Building on this foundation, the research elucidates HSR drivers’ visual characteristics under varying natural lighting scenarios, enhancing the body of knowledge on HSR drivers’ visual behaviors.

The remainder of this paper is structured as follows. Section 2 reviews the related works and presents the hypotheses of this research. Section 3 details the methodology, including the establishment of a general framework to examine the impact of lighting on drivers’ visual behavior and details of specific applications to explore the influence of natural light on HSR drivers’ visual characteristics. Section 4 discusses the data analysis and results, encompassing both natural light environment analysis and eye-tracking metrics analysis. Finally, Section 5 provides the discussion and conclusions, highlighting the practical implications, research limitations, and recommendations for future research.

## 2 Related works and hypotheses

### 2.1 Influence of lighting environment

Due to the critical role of visual information, abundant research has been conducted on the visual behavior of drivers (19, 20). Influence factors include personal ones such as driver’s experience (21), age (22), gender (23), emotional state (24), fatigue levels (25, 26), and sleep duration (27). Environmental factors like lighting conditions (28) also play a role.

The influence of the lighting environment on the driver’s visual behavior has received wide concern and has been extensively investigated (29, 30). Regarding general lighting conditions, some studies have shown that constant shifts in lighting conditions can lead to a driver’s blurred vision or dizziness (31). When driving day to night, the driver’s visual system must adjust its sensitivity to match the light level. Such changes may elevate their cognitive workload and compromise safe driving abilities, observed by Yan et al. (32). Others discussed the influence of lighting on drivers’ visual characteristics under various tunnel environments. For example, Liu (33) proved that the tunnel lighting quality impacts the driver’s comfort which can be reflected via subconscious pupil and eye movement behaviors. Shen et al. (34) discussed that the design of the lighting environment inside the tunnel profoundly impacts on the drivers’ visual system and has a close correlation with driving safety. These investigations primarily focus on road drivers, with a notable scarcity of research on HSR contexts.

Review of limited HSR-related research indicates that drivers’ eye-movement features can provide important information for HSR driving and they are critical for keeping vigilance (35, 36). Dong et al. (17) indicate that visual indicators are vital for drivers’ hazard perception in a high-speed driving task. Scholars also demonstrated that light stimuli will exert intervention on HSR drivers’ visual (15, 37). Visual characteristics are vital for predicting HSR driver’s fatigue (38). Existing research has highlighted that the lighting environment can have an impact on the visual behavior of HSR drivers, particularly under specific conditions such as driver fatigue and hazard perception. This study pertains to more general driving conditions and aims to investigate how variance in natural light influence HSR drivers’ visual behavior.

Besides, as shown in Table 1, lighting indicators namely luminance and illuminance are the most concern by researchers. Some studies also use correlated color temperature to indicate the lighting environment (39–41). Campbell et al. (42) found that higher illuminance levels triggered increased activity in the posterior hypothalamic region, elevating emotional perception and task completion. Bhagavathula et al. (43) show that the visual performance is acceptable between 7 and 10 lx of illuminance, which is an effective strategy to increase visual performance for a wider range of drivers and also be an energy-efficient method. In a study by Hu et al. (39), 12 car drivers participated in an outdoor visual recognition test. The results indicated that illuminance and correlated color temperature significantly influenced drivers’ visual recognition capabilities during nighttime in areas of low meteorological visibility. A higher correlated color temperature light source could enhance the drivers’ visual distance perception.

**Table 1:** Related research.

Reference	Lighting index	Lighting classification	Lighting measurement	Visual index	Visual measurement
Bassani and Mutani (18)	(1) Luminance	Sunny, cloudy, nighttime	(1) Delta ohm hd2302.0 lux meter	(1) Visibility level (vl)	Luminance contrast ratio of a small target
	(2) Illuminance		(2) Derived from illuminance data		
Yoomak and Ngaoptakkul (44)	(1) Average illuminance	Dry road surfaces (r1, r2, r3, r4), wet road surfaces (w1, w2, w3, w4) based on the international commission on illumination	DIALux	(1) Visual performance	Indicated by average luminance
	(2) Average luminance			(2) Visual comfort	
	(3) Overall uniformity				
	(4) Longitudinal uniformity				
	(5) Surround ratio				
	(6) Threshold increment				
He et al. (46)	(1) Luminance	Standard white visual environments and six visual environments with three different luminance levels.	Spectroradiometers	(1) Reaction time	(1) Reaction time
	(2) Correlated color temperature (CCT)			(2) Missed target rate	(2) Missed target rate
Ma et al. (40)	(1) Correlated color temperature	Nine lighting scenarios based on different configurations of CCT (2,000, 4,000, 6,500 k) and illuminance (200, 500, 750 lux)	Autodesk Revit software	Visual perception including	Visual perception questionnaire and the Landolt c test
	(2) Illuminance			(1) comfort	
				(2) naturalness	
				(3) dimness	
				(4) warmness	
Hu et al. (39)	(1) Meteorological visibility	Divided based on meteorological visibility and illuminance level	(1) Forward scattering meteorological visibility meter	(1) Traffic visual distance (TVD)	Outdoor visual recognition tests
	(2) Illuminance	Low illuminance (200–300 lx) and high illuminance (450–500 lx)	(2)(3) Konica Minolta CL-500A spectroradiometer		
	(3) Correlated color temperature				
Kang et al. (7)	(1) Average luminance (Lav)	On basis of the current Chinese tunnel lighting specifications, 1.0, 0.5/0.6/0.7/0.8, etc.	luminance and CCT of adjustable LED lights	(1) Reaction time	(1) Timer
	(2) Luminance longitudinal uniformity (U1)			(2) Pupil area change rate	(2) ASL Model H6 eye-tracking device and EYEPOS, EYENAL software
				(3) Blink frequency	
Liang et al. (5)	(1) Luminance reduction coefficient	Very comfortable, comfortable, uncomfortable, and extremely uncomfortable based on the comfort range of Visual Characteristic Indicators	(1) Digital camera and Spectrascan PR-655 spectral radiance meter	(1) Pupil area change rate	Dikablis Glasses 3 eye tracker
	(2) Road surface illuminance		(2) TES 1339R Data Logger Light Meter Pro illuminance meter	(2) Average saccade speed	
	(3) Visibility		(3) BN-SDTRA10H portable tunnel light transmittance detector		

This research employs illuminance, which is the measure of light flux received per unit area, to represent the natural lighting conditions. In essence, illuminance serves as a reliable indicator of the natural light environment. It reflects the lighting levels within the environment, a critical factor for drivers’ visual perception, which directly impacts the clarity of vision and road safety. Low illuminance levels can lead to increased eye strain, whereas high levels may result in glare. Keeping illuminance within an optimal range helps minimize eye fatigue and stress, thereby improving comfort and safety during extended driving sessions. Existing studies on drivers’ visual behavior typically focus on road driving under various lighting conditions. Our study, however, examines the impact of natural lighting conditions on HSR drivers.

### 2.2 Analysis of light environment

Existing studies abound in examining the impact of the lighting environment on drivers’ emotions, comfort levels, and productivity. It is crucial to quantify and classify the lighting conditions to assess the influence of such environmental factors. As detailed in Table 1, researchers commonly utilize optical devices to gauge lighting parameters, including lux meters (18), spectroradiometers (41), and illuminance meters (18). Additionally, software programs like DIAlux (44) and Autodesk Revit (43) facilitate the simulation and analysis of natural lighting scenarios.

In Table 1, the classification of lighting conditions is explored through three distinct research streams. The first stream draws upon established standards and specifications from various infrastructure sectors, including railways, tunnels, and bridges. Notable references include guidelines from the International Commission on Illumination (CIE) (44), Visual Characteristic Indicators (18), the ‘Railway Lighting Design Code’ (TB10089-2015), China’s highway tunnel lighting standards (15), and Chinese tunnel lighting specifications (7). The second stream utilizes objective natural factors to categorize lighting, such as differentiating between sunny, cloudy, and nighttime conditions (17), day versus night scenarios, and distinctions between tunnel interior and exterior environments. The third stream develops a lighting taxonomy based on previous studies, including parameters like CCT (correlated color temperature) values (2,000 K, 4,000 K, 6,500 K) and illuminance levels (200, 500, 750 lux) (43), as well as categorizing illuminance into low (200–300 lx) and high (450–500 lx) levels (41).

Existing research predominantly concentrates on enhancing driving comfort and safety by optimizing the lighting environment, typically using simplified classifications of lighting conditions. However, there is a growing need for more sophisticated, automated, and intelligent approaches to deepen our understanding of the lighting environment’s impact. Few studies incorporating lighting analysis have employed machine learning techniques. The classification of lighting environments is a clustering problem. Among the prevalent clustering algorithms, K-means, hierarchical clustering, and DBSCAN are extensively utilized. K-means clustering, as a fundamental and widely-used approach, demonstrates adaptive capabilities by dynamically optimizing clustering outcomes based on intrinsic data patterns. In the context of lighting environment analysis and driver behavior, K-means has been utilized for light pollution risk assessment (33), and for categorizing drivers according to their driving styles (7) and habits (5). Therefore, this study employs the K-means clustering algorithm to categorize natural light environments and examines the influence of varying lighting conditions on drivers’ visual behavior.

### 2.3 Drivers’ visual behavior

The visual behavior of drivers is crucial for the safety and efficacy of driving performance, as it is through vision that road conditions are primarily perceived. Researchers have identified various visual metrics to understand drivers’ visual behavior, which are broadly classified into subjective and objective measures. As depicted in Table 1, subjective measures encompass visual comfort, performance, and visibility, typically assessed through questionnaires. For instance, Ma et al. (40) developed nine illuminated vehicle environment (IVE) lighting scenarios along with a questionnaire to gauge drivers’ responses to visual perception aspects such as comfort, naturalness, brightness, and warmth. Liang et al. (5) employed the K-means clustering technique to define the comfort zones for Visual Characteristic Indicators (VCIs), ranging from very comfortable to extremely uncomfortable, and examined the quantitative connections between drivers’ visual traits and tunnel lighting conditions. Leccese et al. (45) conducted experiments that revealed a strong association between an individual’s perception ability and lighting quality.

Objective indicators include the pupil area change rate, saccade speed, and blink frequency, all of which are assessed using an eye tracker. Consequently, eye movements serve as critical metrics for understanding a driver’s visual behavior within a lighting setting (41). Visual attributes can be quantified through eye movement metrics, such as pupil size, fixations, saccades, and blinks (18). The variation in pupil diameter reflects the visual adaptation and the visual demand during driving. Typically, the average rate of change in pupil diameter is employed as a measure to evaluate the quality of the visual lighting environment (46). Fixations and saccades provide essential visual information about the train and driving conditions to the driver (47), and they also indicate the driver’s cognitive workload (48). Number of fixations and saccades are commonly used indicators. Time to first fixation (TFF) denotes the point at which areas of interest (AOIs) come into the driver’s field of view as their gaze first fixates on them (49). This metric can indicate how swiftly a driver can detect an AOI. Hence, in this study, we utilize average pupil diameter (APD), number of fixations (NF), time to first fixation (TFF), and number of saccades (NS) to delineate the visual characteristics of HSR drivers.

It has been observed that lighting levels significantly impact an individual’s visual behavior (41). Elevated lighting conditions have been shown to boost emotional excitement, visual acuity, and the efficiency of task execution (39, 40). Accordingly, we posit that lighting levels have the potential to alter the visual behavior of HSR drivers. Specifically, the diameter of the pupil, which is the central opening in the iris, plays a crucial role in regulating the amount of light that enters the eye. There is a notable correlation between pupil diameter and the level of illuminance, primarily due to the pupil’s automatic adjustment to light intensity. As light intensity rises, the pupil constricts to decrease the influx of light, thereby avoiding discomfort caused by excessive brightness. Conversely, in dim lighting conditions, the pupil dilates to permit more light to enter, thereby enhancing the driver’s ability to detect light and improving visual sensitivity and recognition in dark environments. This leads to the formulation of Hypothesis 1.

**H1**. Low illuminance environment increased HSR drivers’ average pupil diameter.

The fixation and saccade reflect the level of drivers’ visual attention dedicated to various areas within a given environment. These metrics serve as crucial indicators for assessing how visual attention is distributed. They reveal the degree to which drivers concentrate on processing visual data and the amount of visual demand placed upon them. In the presence of complex or unfamiliar visual stimuli, or when encountering abrupt changes in lighting conditions, drivers require increased fixation and saccades to secure essential road information. This allows them to gather more comprehensive data about the road environment, aiding in the identification and assessment of potential risks to inform suitable driving choices. Moreover, under conditions of high illuminance, drivers tend to make their first fixation sooner as they can more readily access external information. Drawing from these analyses, Hypotheses 2, 3, and 4 are proposed.

**H2**. High illuminance environment increased HSR drivers’ number of fixations.

**H3**. Drivers’ time to first fixation occurs earlier in the high illuminance condition.

**H4**. High illuminance environment increased HSR drivers’ number of saccades.

## 3 Methodology

### 3.1 Framework design

**Figure 1:**
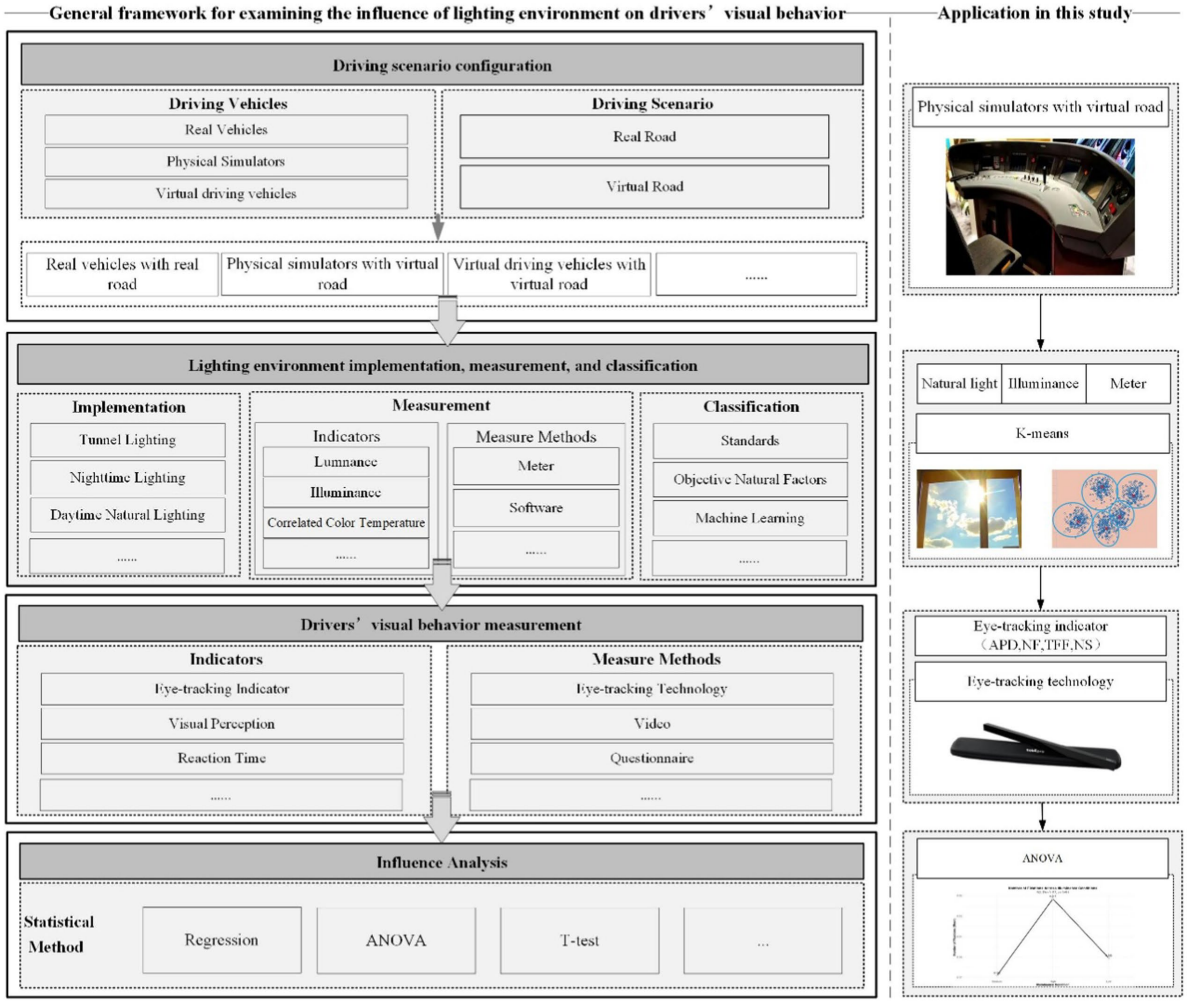
General framework for examining the influence of light environment on drivers’ visual behavior.

Through a comprehensive analysis of existing studies on light environment and drivers’ visual behavior, we systematically reviewed relevant methodologies, techniques, and measurement approaches. This allows us to establish a methodological framework for examining the influence of lighting environment on drivers’ visual behavior, as illustrated in Figure 1. Furthermore, we apply this framework to explore natural light environment on HSR drivers’ visual behavior.

The general framework involves four main steps. The initial step includes setting driving scenario, which encompasses the selection of driving vehicles and scenes. Taking train driving for example, potential driving vehicles include real train, train simulator, and virtual driving vehicles (e.g., driving simulation game). Driving scenarios involve real or virtual scenes. While real-world train operations in actual environments provide the most authentic data, their implementation is often constrained by safety considerations and stringent operational protocols. Consequently, simulator-based or virtual driving environments are more frequently employed in research settings. The second step focuses on the implementation, measurement and classification of lighting environment. Lighting environment implementation involves deploy specific light conditions, such as tunnel lighting or nighttime conditions. Measurement of lighting environment requires to identify indexes to quantify lighting parameters. Subsequently, the classification of lighting environments facilitates more detailed analysis of the influence on drivers’ behavior.

The third step aims to measure drivers’ visual behavior through the selection of relevant indicators and appropriate measurement method. Visual indicators typically include eye-tracking indicators, visual perception metrics (e.g., visual comfort), and response time. Eye-tracking devices, video recording systems, and questionnaire are common measure approach. The fourth step is statistical analysis of lighting environment effects on visual behavior by aid of statistic methods such as regression, ANOVA, and other relevant statistical techniques.

### 3.2 Driving scene

**Figure 2:**
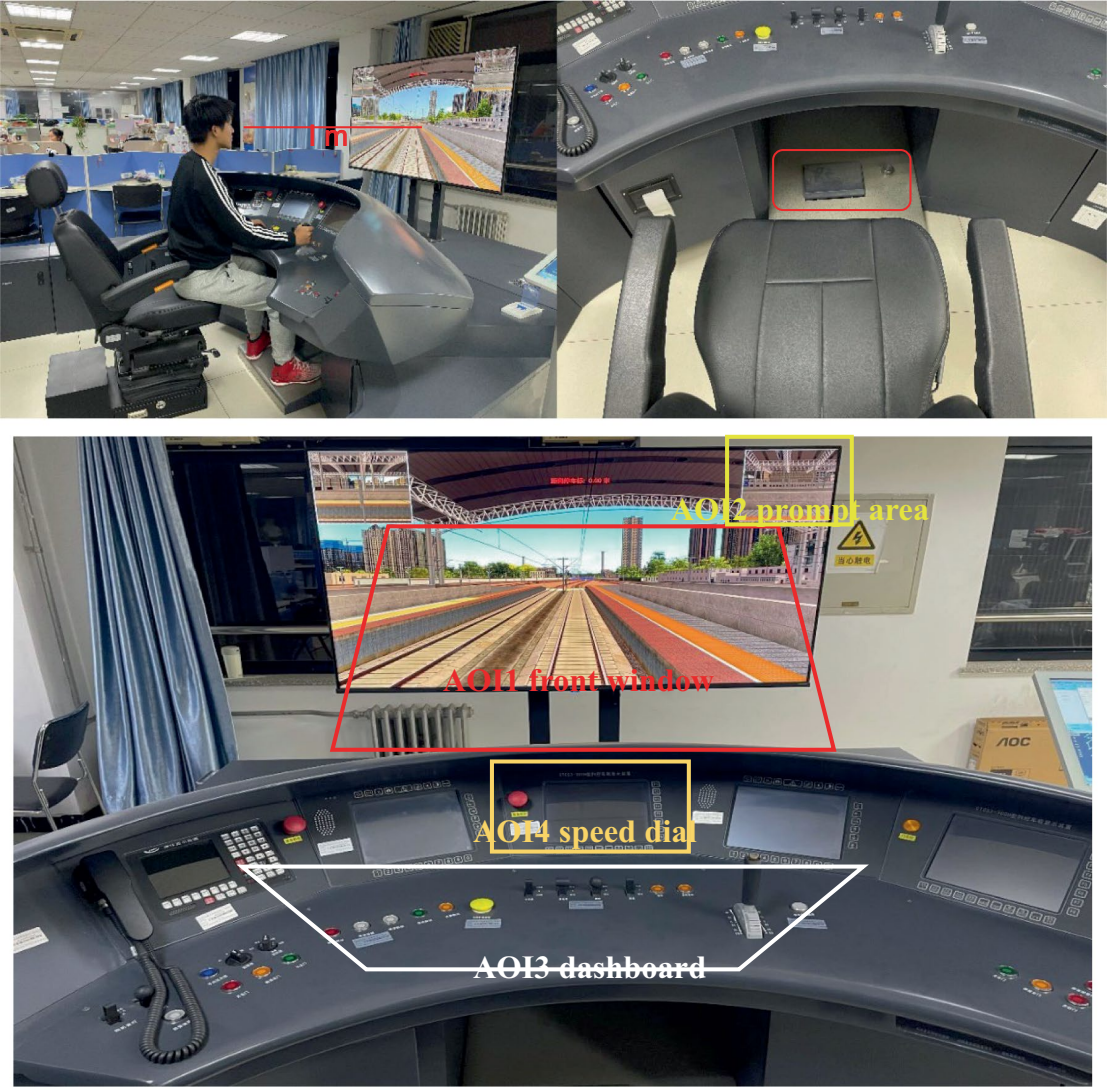
The apparatus and the experimental situation.

The Fuxing HSR simulator system is used in this study, as shown in Figure 2. It contains Fuxing simulator and several authentic HSR lines in China. The simulator is one-to-one simulation driving equipment and its internal is completely consistent with the real Fuxing HSR, which is equipped with a dashboard, foot pedal, brake button, etc. The system has a 55-inch 1080p LCD monitor that is used to display the virtual scene with a display size of 122 cm wide and 70 cm high. The simulator comes with a real driver seat, the seat is adjustable to make sure the participants can find the most comfortable sitting position and ensure the distance between the monitor and the eyes of the participants is about 1 m.

Authentic HSR lines include long and short tunnels, viaducts, and other designs. Weather designs are also available, such as sandstorms, cloudy and rainy days, tornadoes, and nights. In this study, we choose the lines from Zhengzhou, Henan Province to Beijing as our main experimental route (including tunnels on the way). In the experiment, drivers were able to experience a completed driving process, including leaving the station, driving along the route, and stopping at the next station. During the driving, the train’s speed could vary according to the external environment. The drivers need to control the speed to avoid exceeding the limit. Upon reaching the next station, drivers were required to position the train correctly. The experiment concluded after this.

### 3.3 Natural light environment

This study focuses on natural light environment, defined as the light conditions occurring from sunrise to sunset, exclusively considering sunlight-derived illuminance, while excluding artificial lighting systems. The driving simulator was positioned in front of the window to ensure direct exposure to natural light conditions. Referring to previous research, illuminance was employed as the metric to quantify light environment. The PR-300YM-4G illuminance meter, paired with the Prsen RS485 photosensitive fatigue sensor, was employed to gauge the level of illuminance. It updates illuminance data per minutes.

In this study, we utilize unsupervised machine learning methods for the automated classification of light environment. As an attempt to apply machine learning in this domain, we use K-means algorithm, a fundamental and widely-used clustering approach. K-means enables the intelligent partitioning of complex data into distinct clusters, thereby facilitating the identification and interpretation of characteristic patterns within each group. When implementing K-means algorithm, the Elbow Method (48) was used to determine the optimal number of clusters (*k*) by calculating the sum of squared errors (SSE) within clusters for different *k* values. The *k* value corresponding to the elbow point, where the SSE significantly decreases, was chosen. Once the optimal *k* was determined, the K-means algorithm was applied to group the illuminance data. The algorithm followed these iterative steps:

Step 1: Randomly initialize *k* cluster centroids $\left(\mu_{1},\mu_{2},\cdots ,\mu_{k}\right)\text{.}$

Step 2: Assign each data point to the nearest cluster centroid. The distance between a data point *x* and a centroid $\mu_{i}$ was calculated using the Euclidean distance formula:


(1) $$||x-\mu_{i}||=\sqrt{\sum_{j=1}^{n}{\left(x_{j}-\mu_{ij}\right)}^{2}}$$

Step 3: Recalculate the centroid of each cluster based on the current members of the cluster. The new centroid $\mu_{i}$ is the mean of all points (*x*) in cluster *i*:


(2) $$\mu_{i}=\frac{1}{\left|C_{i}\right|}\sum_{x\in C_{i}}x$$

The assignment and updating steps were repeated until the cluster centers no longer showed significant changes or until a preset number of iterations was reached.

### 3.4 HSR drivers’ visual behavior

Eye-tracking technology was used to indicate and measure drivers’ visual behavior. As mentioned before, average pupil diameter (APD), number of fixations (NF), time to first fixation (TFF), and number of saccades (NS) were selected to reflect drivers’ visual characteristics in term of pupil, fixation and saccade. The Tobii Nano eye-tracker, boasting a sampling rate of 1,200 Hz, was used to record the visual attention patterns of HSR drivers. This eye-tracking system is non-invasive and operates on a video-based platform. It leverages corneal reflection and the pupil’s center as key features to monitor eye movements.

### 3.5 Data collection

The research focuses on the impact of natural light environment on drivers’ visual behavior. It was approved by the Ethics Committee of Economics and Management at Beijing Jiaotong University, which was conducted in accordance with the local legislation and institutional requirements. The illuminance data were recorded by an optical device with a sampling interval of 1 min from 6 am to 6 pm daily between November 30 and December 6, 2023 (7 days), yielding 5,040 illuminance samples. Regarding collecting visual data, the experimental sessions were scheduled from 8 am to 6 pm with a 30-min period for each participant, resulting in 20 distinct sampling periods per day.

Due to challenges in recruiting HSR drivers, the study employed student participants from a Chinese public university with strong railway affiliations. To enhance the validity of the study, students majoring in railway transportation were subjected to targeted recruitment, ensuring that participants have fundamental theoretical knowledge of railway operations. Additionally, a comprehensive simulator training with three-day practice was conducted, followed by an examination to verify participants’ competence in operating the Fuxing HSR simulator. From an initial pool of 50 registered trainees, 35 participants successfully completed the training and passed the examination. The final participants, aged between 18 and 23 years, have normal or corrected-to-normal vision. The training program ensured participants’ familiarity with HSR simulator operations and their understanding of HSR driving regulations and procedures.

Participants can select any 30-min period between 8 am and 6 pm for their experiment session. Before the experiment began, participants were informed of the experiment procedure and provided written informed consent. Upon completion, participants were compensated with 30 RMB for their time. Following data quality assessment, three participants were excluded due to incomplete driving tasks, and four were removed due to eye-tracking data validity rates below 70%. Consequently, the final dataset included valid eye-tracking data from 28 participants, yielding an overall validity rate of 80%.

## 4 Data analysis and results

### 4.1 Natural light environment analysis

2,817 lighting data relevant to the train driving experiment was selected for further analysis. In this study, light data preprocessing was first conducted by removing outliers and eliminating missing values and extreme values to avoid any potential impact on the experimental results. The descriptive statistical result of the illuminance data is presented in Table 2.

**Table 2:** Descriptive statistics results.

	Count	Mean	Std	Min	25%	50%	75%	Max
Illuminance	2,817	5,258.314	8,192.988	3	625	2,515	5,479	48,162

#### 4.1.1 Trend and time series analysis

**Figure 3:**
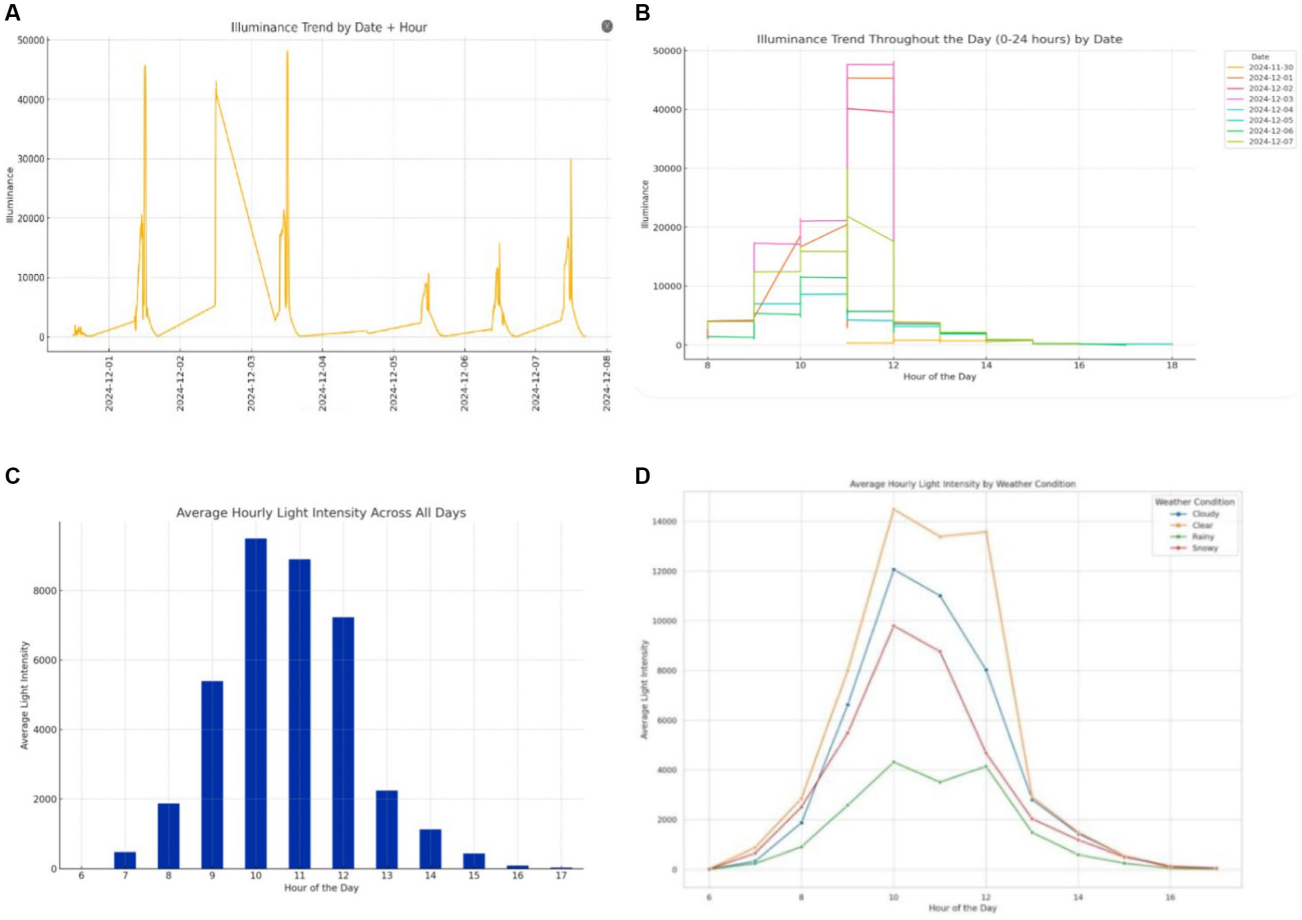
Results of trend and time series analysis. **(A)** Illuminance trend by date and hour; **(B)** illuminance trend throughout the day; **(C)** hourly average illuminance; **(D)** hourly average illuminance by weather category.

We visualize the data to further explore the patterns of change in illuminance. A line chart was first created, as shown in Figure 3A. It indicates that illuminance exhibits a regular variation over a day, changing systematically with time. Subsequently, the data variation was plotted on a graph with hours as the horizontal axis to further observe the trend in illuminance changes, as shown in Figure 3B. The graph illustrates that starting from 8 _am_ each day, the illuminance begins to rise, reaching its peak around 11 _am_ to 12 _pm_, after which it starts to decline.

To gain a clearer understanding of the trend, we computed the hourly mean for each day and subsequently compiled these daily averages to obtain data for corresponding periods across multiple days. This method yielded more consistent and indicative illuminance measurements. Figure 3C further validates the inferences drawn previously. Moreover, in alignment with Bassani and Mutani (18), we took into account the possible variations in illuminance caused by different weather conditions. Consequently, the data gathered from November 30 to December 18 was sorted into four categories based on weather patterns: sunny, cloudy, rainy, and snowy, as depicted in Figure 3D.

According to Figure 3D, we observe that on sunny days, the illuminance is higher, especially around midday. This higher level of illuminance can lead to notable glare issues, posing a major challenge for drivers operating precision control systems. On cloudy days, while the illuminance is lower than on sunny days, it still exhibits considerable fluctuations, particularly during the transition from morning to afternoon. These fluctuations can cause the lighting conditions within the cabin to vary constantly, potentially affecting the driver’s visual adaptation. On overcast or rainy days, the illuminance is generally lower, necessitating sufficient artificial lighting within the cabin to compensate for the lack of natural light. In low-illuminance environments, the difficulty of visual recognition increases, which could impact the driver’s ability to quickly identify and respond to information. The illuminance on snowy days is slightly lower than on cloudy days, with a similar overall trend.

#### 4.1.2 K-means clustering analysis

##### 4.1.2.1 General analysis

**Figure 4:**
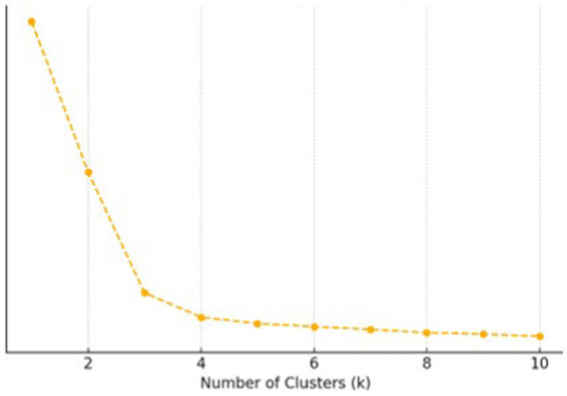
Elbow method for K-means.

**Figure 5:**
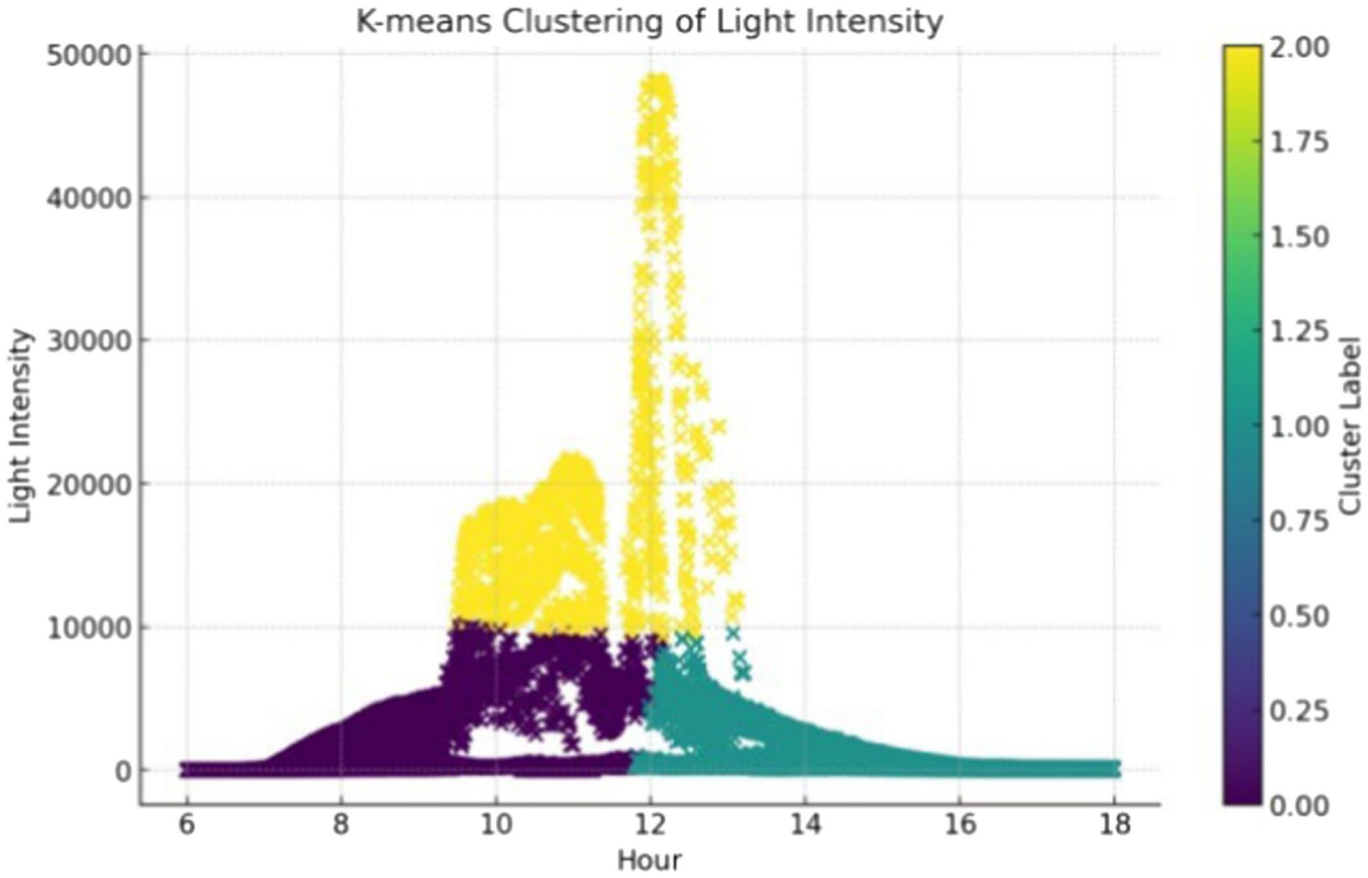
Illuminance clustering results based on K-means.

Given that illuminance data may vary with time or environmental conditions (weather, road conditions, and time periods), K-means clustering analysis was employed to analyze further driving behavior. First, the data was cleaned by removing any outliers (e.g., erroneous extreme illuminance readings) and missing values. The data was then standardized or normalized to reduce the impact of different units of measurement. Next, *k* = 3 was determined by using the Elbow method, illustrated in Figure 4. After iterations, the calculation of clustering by using Equations 1, 2, and the clustering results for illuminance data are shown in Figure 5.

- **Cluster 0:** This cluster primarily includes the afternoon period (approximately 2 _pm_ to 5 _pm_), with an average illuminance of 1,043 lux, and a fluctuation range from 0 to approximately 5,962 lux. This cluster represents the lower and more stable illuminance levels in the afternoon, likely due to the sun’s lower position, resulting in reduced light intensity.
- **Cluster 1:** This cluster encompasses the morning period (approximately 9 _am_ to 12 _pm_), with an average illuminance of 12,612 lux, making it the cluster with the highest light intensity. The fluctuation range is from 6,820 to 22,918 lux. This indicates the most intense lighting conditions in the morning, with a sharp increase in illuminance reaching its peak. Special attention is needed to avoid visual discomfort or driving interference due to strong light.
- **Cluster 2:** This cluster covers the early morning to late morning period (from around 6 _am_ to 11 _am_), with an average illuminance of 1,247 lux and a fluctuation range from 0 to approximately 6,295 lux. This cluster reflects the gradually increasing light intensity from early morning to late morning, which could affect driving conditions during the morning rush hour.

##### 4.1.2.2 Further analysis

As mentioned above, weather is an important factor that influences illuminance. Figure 3D indicates that illuminance on rainy days is lower than that on other days. Therefore, further K-means analysis was conducted based on different weather conditions, namely rainy days and other days (i.e., sunny days, cloudy days, and snowy days).

**Figure 6:**
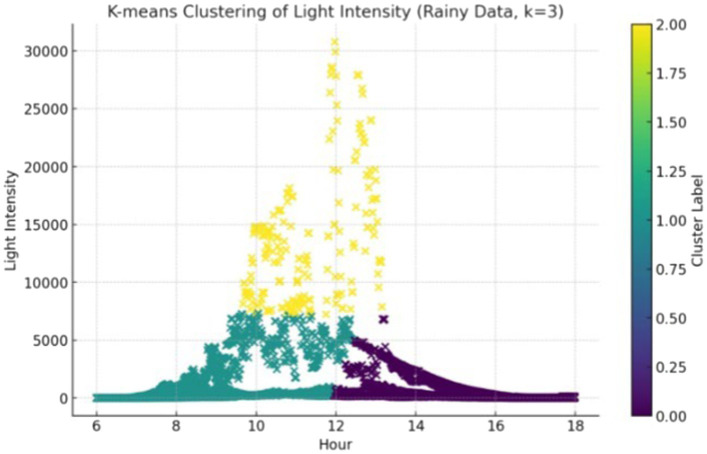
Rainy day illuminance clustering results based on K-means.

For rainy day (Figure 6), Cluster 0 is typified by low levels of illuminance, with values predominantly on the lower end and sunlight insufficiently strong. Cluster 1 shows a steady rise in sunlight in the morning, concurrent with rainy weather. For Cluster 2, the illuminance data reach their highest points, indicating that even when clouds are present, the lighting conditions remain fairly favorable.

**Figure 7:**
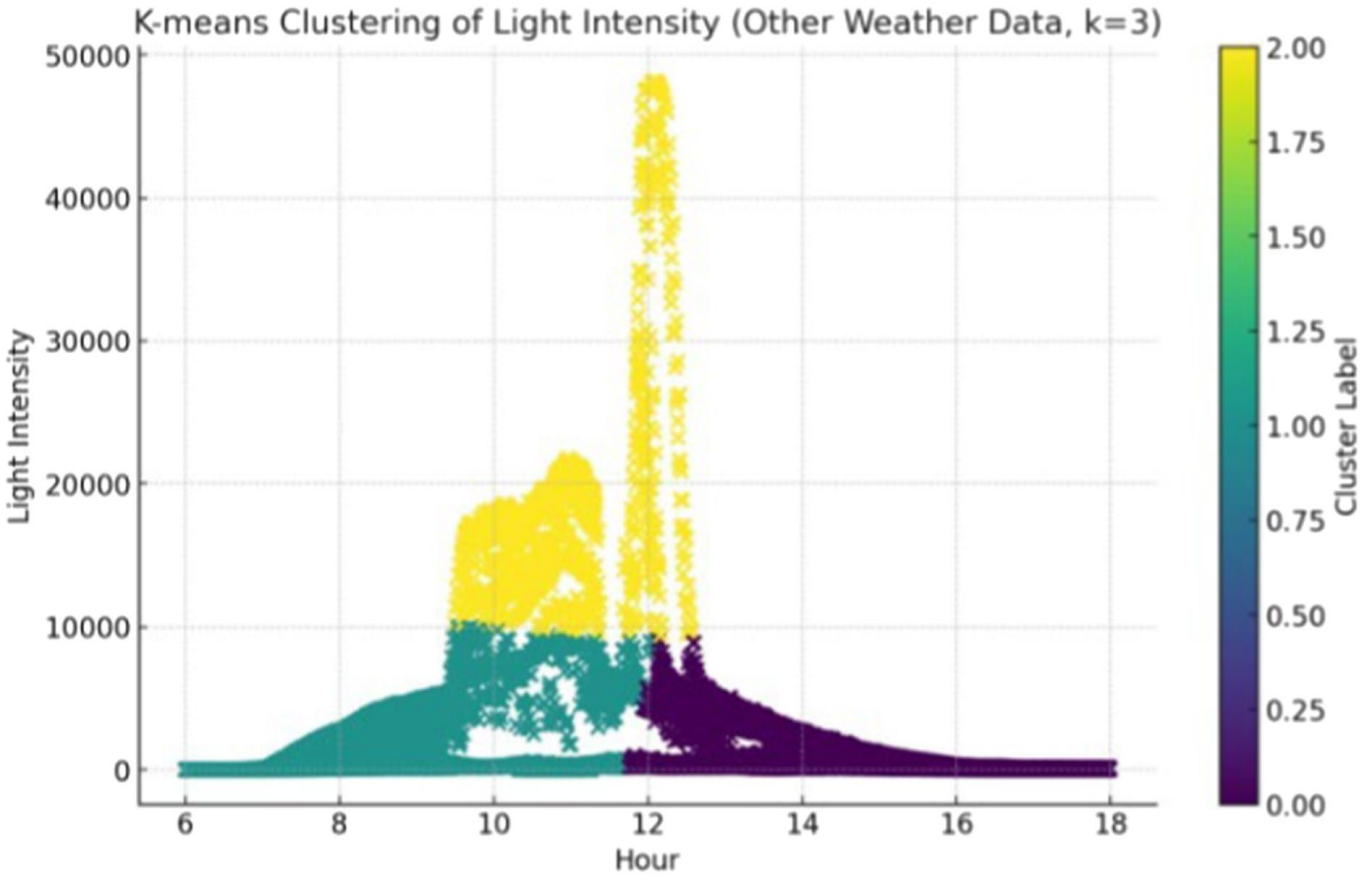
Other day illuminance clustering results based on K-means.

The K-means clustering algorithm was similarly employed to analyze other weather conditions (Figure 7), resulting in three primary categories as depicted in Table 3. Cluster 0 denotes the times with consistently lower levels of illuminance, during which the ambient light is predominantly composed of scattered and reflected light. Cluster 1 corresponds to moderate illuminance levels, reflecting the morning’s gradual sunlight increase. Cluster 2 signifies the times with the highest illuminance of the day, coinciding with the peak midday sunlight.

**Table 3:** Further K-means analysis based on rainy days and other days.

Cluster		Time	Data range
Cluster 0	Rainy	1 _PM_–6 _PM_	0–6,826 lux
(Low)	Other	Around 1 _PM_	0–9,028 lux
Cluster 1	Rainy	Around 9 _AM_	0–7,307 lux
(Medium)	Other	Around 9 _AM_	0–10,060 lux
Cluster 2	Rainy	10 _AM_–1 _PM_	7,225–30,776 lux
(High)	Other	9 _AM_–1 _PM_	9,406–48,162 lux

##### 4.1.2.3 Results

Initially, qualitative analyses of the illuminance were conducted through descriptive statistics, revealing that the illuminance fluctuates most noticeably between 9 _am_ and 1 _pm_, with peaks occurring during this period. Subsequently, the data was further segmented based on the weather conditions of the day, including four categories (sunny days, rainy days, cloudy days, and snowy days). Visualization of the data revealed obvious differences in the fluctuation patterns between the rainy days group and the other three groups. Therefore, the data was segmented further for subsequent clustering analysis to explore the fluctuation patterns of illuminance throughout the day.

The analysis indicated that although there were distinctions between the rainy-day group and the other groups, the clustering outcomes continued to segment the data into three distinct clusters—low illuminance, medium illuminance, and high illuminance—mirroring the clustering pattern observed in the other groups, albeit within varied data ranges. Therefore, based on the validation of descriptive statistics and clustering analysis, appropriate clustering of illuminance covers three clusters.

(1) Medium illuminance (6 _am_–9 _am_)

This phase occurs from 6 _am_ to 9 _am_ in the morning. During this period, the illuminance gradually increases from the extremely low levels of the night, influenced by the angle of the rising sun. The illuminance shows a stable upward trend.

(2) High illuminance (9 _am_–1 _pm_)

This phase occurs from 9 _am_ to 1 _pm_ and represents the period with the strongest illuminance throughout the day. During this time, the illuminance rises sharply and reaches its peak, which can easily cause glare.

(3) Low illuminance (1 _pm_–6 _pm_)

This phase occurs from 1 _pm_ to 6 _pm_. During this period, the illuminance gradually decreases, entering a slow decay phase in the afternoon. As the sun’s angle lowers, direct sunlight diminishes, and the illuminance is primarily composed of scattered light.

### 4.2 Eye-tracking metrics analysis

Based on the K-means clustering results, the lighting environment was divided into three categories: the low illuminance group [3], the medium illuminance group [1], and the high illuminance group [2], corresponding to the periods of 1 _pm_–6 _pm_, 6 _am_–9 _am_ and 9 _am_–1 _pm_, respectively. Besides, considering two tunnels in our experiment lines, we also consider the tunnel scenario. Further, referring to the driving simulation scenario and driving task, four areas of interest (AOIs) are divided, AOI1 front window, AOI2 prompt area, AOI3 dashboard, and AOI4 speed dial, as shown in Figure 1. Based on data from 28 valid participants, ANOVA with 3 lighting environments * 2 tunnels * 4 AOIs was conducted to analyze the drivers’ visual characteristics under natural light conditions.

#### 4.2.1 Average pupil diameter (APD)

**Figure 8:**
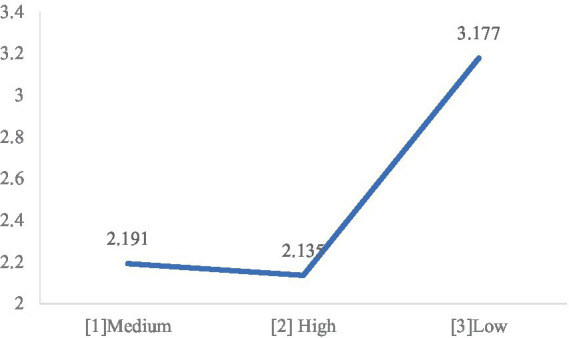
Average pupil diameter under light conditions.

The variation in pupil diameter was statistically significant across the three illuminance conditions (*F* (2, 25) = 9.909, *p* < 0.001). Further analysis revealed that the APD in the low illuminance group [3] was significantly higher than those in the medium [1] and high [2] groups (*M*_[1]_ = 2.191, *M*_[2]_ = 2.135, *M*_[3]_ = 3.177, *p*_[1][3]_ = 0.002, *p*_[2][3]_ = 0.001). In contrast, the difference between the medium and high illuminance groups was insignificant, as shown in Figure 8. Hence, H1 was supported.

#### 4.2.2 Number of fixations (NF)

**Figure 9:**
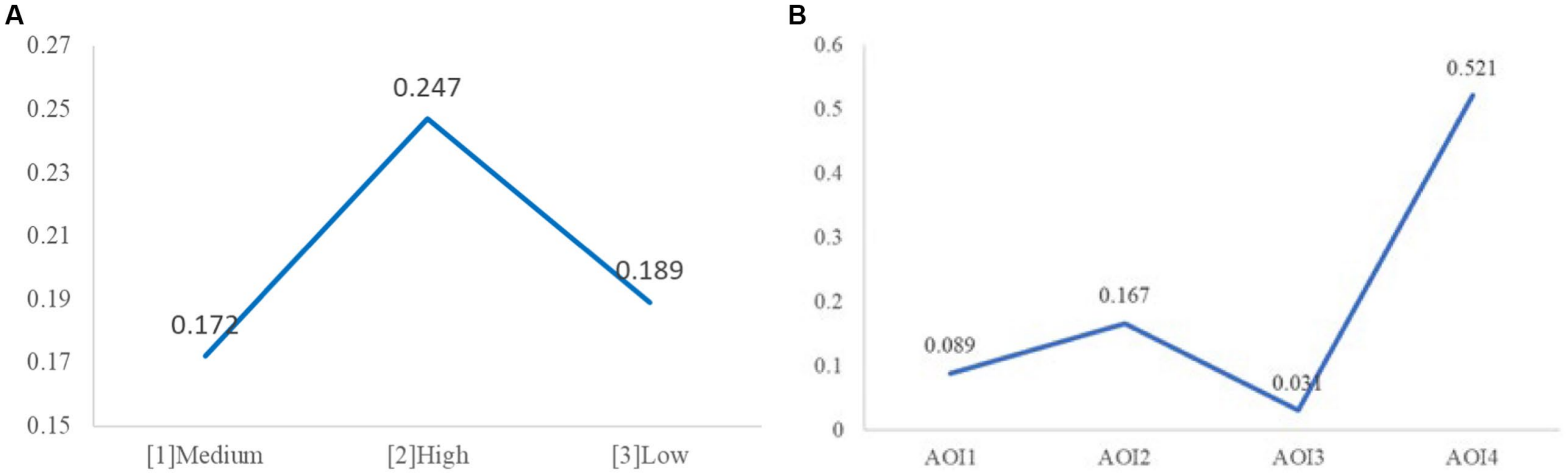
**(A)** Number of fixations under light conditions. **(B)** Number of fixations under AOIs.

The analysis of the number of fixations (NF) revealed that the main effect of illuminance was marginally significant (*M*_[1]_ = 0.172, *M*_[2]_ = 0.247, *M*_[3]_ = 0.189, *F* (2, 25) = 3.127, *p* = 0.061). As shown in Figure 9A, further analysis showed that the NF in the high illuminance group was significantly higher than that in the medium (*p*_[2][1]_ = 0.028) and low (*p*_[2][3]_ = 0.048) group, with no significant differences observed in other scenarios. Hence, H2 was supported.

For tunnel scenes, its main effect was not significant (*F* (1, 25) =1.914, *p* = 0.179), while the main effect of AOI was significant (*F* (3, 23) = 78.096, *p* < 0.001). Further analysis (as shown in Figure 9B) revealed that the speed dial AOI (*M*_AOI4_ = 0.521) attracted the most fixations, significantly more than the other three AOIs (*p*_AOI4-AOI1_ < 0.001, *p*_AOI4-AOI2_ < 0.001, *p*_AOI4-AOI3_ < 0.001). The prompt area AOI (*M*_AOI2_ = 0.167) was the second most fixated, significantly more than the other two AOIs (*p*_AOI2-AOI3_ < 0.001, *p*_AOI2-AOI1_ < 0.001). Following were the front window (*M*_AOI1_ = 0.089) and the dashboard (*M*_AOI3_ = 0.031), with a significant difference between the two (*p*_AOI1-AOI3_ < 0.001).

#### 4.2.3 Time to first fixation (TFF)

**Figure 10:**
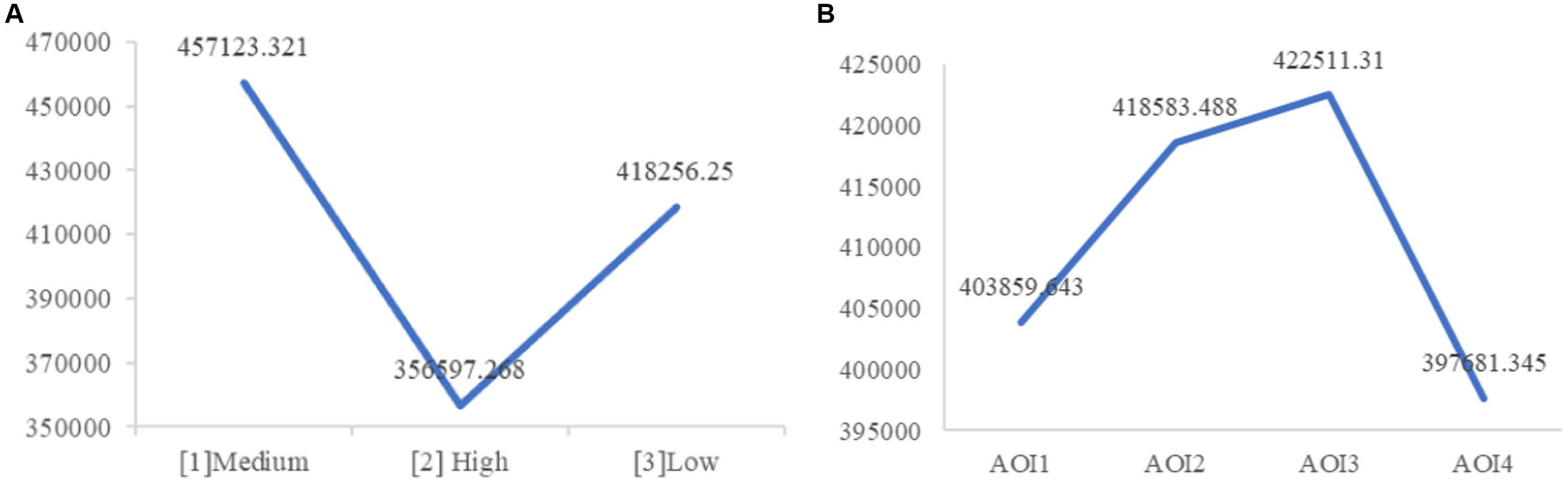
**(A)** Time to first fixation under light conditions. **(B)** Time to first fixation under AOIs.

The analysis of the time to first fixation revealed a significant main effect of illuminance (*F* (2, 25) = 3.79, *p* = 0.037). As illustrated in Figure 10A, further analysis indicated that participants in the high illuminance group had a significantly shorter TFF compared to the medium group (*M*_[2]_ = 356,597.268, *M*_[1]_ = 457,123.321, *p*_[2][1]_ = 0.012) and showed a marginally significant difference compared to the low light intensity group (*M*_[3]_ = 418,256.25, *p*_[2][3]_ = 0.066), while the difference between the low and medium light intensity groups was not significant. This suggests that participants in the high illuminance condition formed fixations earlier, hence H3 was supported.

Regarding tunnel scenes, there was a significant main effect (*M*_[1]_ = 253,491.815, *M*_[2]_ = 567,826.077, *F* (1, 25) = 582.016, *p* < 0.001), with participants forming fixations earlier outside the tunnel. This is because the scene outside the tunnel appears earlier. Additionally, the main effect of AOI was significant (*F* (3, 23) = 6.689, *p* = 0.002). Further analysis (as shown in Figure 10B) revealed that the speed dial AOI (*M*_AOI4_ = 397,681.345) received the earliest first fixation, significantly earlier than the other three AOIs (*p*_AOI4-AOI1_ = 0.024, *p*_AOI4-AOI2_ = 0.003, *p*_AOI4-AOI3_ = 0.02). The front window (*M*_AOI1_ = 403,859.643), prompt area (*M*_AOI2_ = 418,583.488), and dashboard (*M*_AOI3_ = 422,511.310) followed. Specifically, the TFF for the front window AOI was significantly earlier than the prompt area AOI (*p*_AOI1-AOI2_ = 0.035), but not significantly different from the dashboard AOI (*p*_AOI1-AOI3_ = 0.09), and the prompt area AOI was not significantly different from the dashboard AOI. This is consistent with the simulated driving task, as participants needed to observe the speed dial after starting the simulation to ensure proper vehicle operation. The TFF for the front window was earlier than the prompt area because participants needed to observe the driving environment through the front window.

#### 4.2.4 Number of saccades (NS)

**Figure 11:**
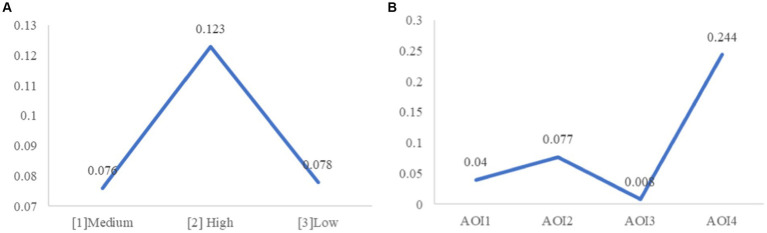
**(A)** Number of saccades under light conditions. **(B)** Number of saccades under AOIs.

The analysis of the number of saccades revealed a significant main effect of illuminance (*F* (2, 25) = 3.38, *p* < 0.05). As illustrated in Figure 11A, further analysis indicated that participants in the high illuminance group had significantly more saccades compared to those in the medium and low groups (*M*_[2]_ = 0.123, *M*_[1]_ = 0.076, *M*_[3]_ = 0.078, *p*_[2][1]_ = 0.039, *p*_[2][3]_ = 0.023), while the difference between the low and medium light intensity groups was not significant. This suggests that participants in the high illuminance condition had more frequent saccades, so H4 was supported.

For tunnel scenes, its main effect was not significant (*F* (1, 25) = 3.419, *p* = 0.076), while the main effect of AOI was significant (*F* (3, 23) = 46.228, *p* < 0.001). Further analysis (as shown in Figure 11B) revealed that the speed dial AOI (*M*_AOI4_ = 0.244) had the most frequent saccade, significantly more than the other three AOIs (*p*_AOI4-AOI1_ < 0.001, *p*_AOI4-AOI2_ < 0.001, *p*_AOI4-AOI3_ < 0.001). The prompt area (*M*_AOI2_ = 0.077), front window (*M*_AOI1_ = 0.040), and dashboard (*M*_AOI3_ = 0.008) followed. Among these, the prompt area AOI had significantly more saccades than the front window AOI and dashboard AOI (*p*_AOI2-AOI3_ < 0.001, *p*_AOI2-AOI1_ < 0.001), and the front window AOI had significantly more saccades than the dashboard AOI (*p*_AOI1-AOI3_ < 0.001).

## 5 Discussion and conclusion

This study examines HSR drivers’ visual characteristics in a natural light environment. A general framework to examine lighting on drivers’ visual behavior was established. This framework encompasses four key components: (1) driving scenario configuration, (2) lighting environment implementation, measurement, and classification, (3) drivers’ visual behavior measurement, and (4) influence analysis. The practical application of this framework to investigate the effects of natural light on HSR drivers’ visual characteristics was detailed. Particularly, an unsupervised machine learning method was used to evaluate and categorize lighting conditions. Specifically, based on illuminance, K-means clustering was adopted to classify the daily light environment. Further, the average pupil diameter (APD), number of fixations (NF), time to first fixation (TFF), and number of saccades (NS) were selected to analyze the driver’s visual behavior under three illuminance groups. Moreover, the visual characteristics of the area of interest (AOIs) were analyzed. The main conclusions are as follows:

First, the unsupervised machine learning method is feasible to analyze and categorize lighting environments. K-means clustering was utilized in this study, and three clusters, low illuminance (1 _pm_–6 _pm_), medium illuminance (6 _am_–9 _am_), and high illuminance (9 _am_–1 _pm_), were identified. The clustering results are acceptable and accountable.

Second, the driver’s average pupil diameter changes with varying illuminance. Literature has manifested that pupil-related indexes can indicate luminance differences in lighting environment (9). Specifically, the diameter of the pupil adjusts spontaneously in response to variations in light intensity, narrowing in well-lit settings and widening in darker ones. Our results are in agreement with earlier studies. When comparing low illuminance levels to those of medium and high intensity, we observe that the decreased luminance in low-light conditions results in a noticeable expansion of the pupil diameter. This indicates that changes in luminance within the low illuminance group are substantial enough to elicit the pupil’s adaptive reaction.

Third, high illuminance enhances HSR drivers’ visual behavior. Specifically, HSR drivers exhibit an increased number of fixations and saccades under high illuminance conditions. This could be attributed to the stimulating effect of bright lighting, which enhances alertness and engagement. This aligns with earlier research indicating that heightened illumination can lead to improved task performance and visual behavior among drivers (%^39^–^41^). Drivers who are stimulated demonstrate an increase in fixations and more frequent eye movements, enabling them to be more vigilant and attentive to their environment, thereby lowering the likelihood of accidents. Furthermore, the quicker initial fixation in brighter conditions reinforces this observation. Due to enhanced processing and elevated alertness, drivers under high-stimulus conditions can zero in on targets swiftly and effortlessly.

Fourth, besides being affected by the lighting environment, the driving task is identified as one of the most important factors affecting HSR drivers’ visual characteristics. Participants in the driving task are required to maintain appropriate speed levels, avoiding any instances of over-speeding. Consequently, the speed dial AOI (Area of Interest) attracts the highest number of fixations and saccades and the earliest initial fixation compared to the AOIs of the prompt area, front window, and dashboard. This indicates that HSR (High-Speed Rail) drivers allocate greater attention, engage in more frequent scanning, and focus on the speed dial sooner to optimize their task performance. Furthermore, in the context of tunnel environments, there were no significant variations observed in the number of fixations and saccades. This could be attributed to the fact that the tunnel scenario was not manipulated to reflect real-world conditions, hence the tunnel did not significantly impact the drivers’ visual behavior.

### 5.1 Practical implications

First, the outcomes of this research establish a methodological framework for examining HSR drivers’ visual characteristics under varying light conditions. The classification of daily light environments facilitates a detailed investigation into HSR drivers’ visual behavior. To maintain drivers’ better visual acuity and ensure driving safety, HSR operating entities can utilize method delineated in this study to access the impact of varying lighting conditions on drivers’ fixation patterns and visual attention. The application of unsupervised machine learning for light environment analysis enables these organizations to automatically quantify and categorize lighting at any given time, across various climates and regions. The approach to analyzing HSR drivers’ visual behavior, supported by eye-tracking technology, offers insights into focal points of drivers’ attention under different lighting scenarios and their adaptation of visual search strategies in response to changing light conditions. For example, by scrutinizing drivers’ fixation points and pupil dilation, etc., managers can formulate more accurate recommendations for improving lighting design, particularly in challenging driving environments such as tunnels, bends, and viaducts.

Second, the findings of this study offer valuable insights for the development of tailored training programs for HSR drivers, aimed to enhance their visual search capabilities and driving safety. Our research indicates that high illuminance levels (9 am to 1 pm) can trigger more activated visual behavior in HSR drivers. This heightened state of attention and vigilance toward the surroundings can improve task performance and reduce accident risk. However, prolonged visual activity may hasten the onset of driving fatigue. Conversely, during periods of low illuminance (1 pm to 6 pm), HSR drivers may experience a decline in attention and alertness, which could adversely affect their task performance. Low illuminance can also induce pupil dilation, potentially resulting in increased visual strain. Consequently, the design of the training programs should consider the distinct visual demands placed on drivers in various driving environments, with particular attention to those who are more prone to accidents (47). For example, novice drivers, aimed to effectively cultivate their attention allocation skills and develop the necessary attention capacity for driving, are better to undergo training during high illuminance (9 am to 1 pm) condition when people are triggered more activated visual behavior. Regarding to training drivers to be more experienced and sophisticated, it is crucial to include attention training under adverse conditions. Training under low illuminance conditions (1 pm to 6 pm) is essential, as in this scenario drivers are more susceptible to reduced attention and alertness, as well as visual fatigue and strain. These insights lay a robust groundwork for further exploration into human-machine-environment interactions, particularly in assessing the engagement of HSR drivers under varying lighting conditions to keep drivers safe driving.

Thirdly, the findings of this study provide significant insights for the development of a HSR driver fatigue monitoring system, based on the evaluation of physiological indicators. According to Healey and Picard (50), fixation acts as an indirect indicator of a driver’s mental state and fatigue level, with a tendency for fixation duration decrease as fatigue levels rise during prolonged driving sessions. Furthermore, Schmidt et al. (51) have identified pupil response as a crucial physiological indicator of increased sympathetic nervous system activity. A study conducted using a driving simulator revealed that drivers exhibit significant variations in heart rate and pupil diameter when experiencing discomfort during driving (52). Consequently, the eye movement characteristics of HSR drivers, as delineated in this study, can be instrumental in setting the appropriate physiological indicators for a fatigue monitoring system.

### 5.2 Limitation and future research

Driving scenario configuration is important in investigation drivers’ behavior. Driving simulation experiments are commonly used in driving-behavior research, primarily owing to their advantages in variable control and scenario customization (53). However, limitations still exist. First, the challenge of replicating the true speed of HSR and the authentic experience of piloting an HSR accurately in a simulator is significant. The findings have not been validated in real-world driving scenarios. Hence, effectiveness of the lighting group and visual characteristics should be further evaluated in the real HSR driving environment to enhance the applicability of the results. Second, the sample population was restricted to students, primarily composed of younger individuals proficient in basic driving abilities and satisfying the basic qualifications for HSR drivers. It is evident that there are differences between experienced drivers and students. Additionally, the sample size is limited. To bolster the credibility of the research findings, subsequent studies should consider to expand the sample size and age range, if possible, to incorporate actual HSR drivers into the participant group. Adopting these methodological enhancements will help ensure a more accurate affirmation of the study’s results.

The field of lighting analysis presents numerous opportunities for future research. First, the exploration of alternative machine learning methods warrants further investigation. This study regards the classification of lighting environment as a clustering problem and demonstrates the effectiveness of K-means algorithm. Depending on specific data characteristics and analytical requirements, future research could expand the methodological scope. For example, consider other unsupervised methods, including other clustering algorithms, association rule learning, and neural network approaches, or supervised approaches such as support vector machines, random forests, and gradient boosting trees, for lighting analysis. Second, future studies could focus on more sophisticated, quantitative, and comprehensive analysis of lighting. This study primarily aims to elucidate the trend in visual behavior changes under varying light environment, and as such, the quantitative findings are somewhat constrained. Building upon the insights gained, future research could delve into more quantitative explorations. This may include determining the threshold for comfortable natural light conditions conductive to HSR driving, identifying the range of lighting conditions that significantly alter drivers’ visual behavior, and quantifying the extent of changes in drivers’ visual responses corresponding to each unit variation in lighting conditions, among other potential avenues of investigation. Third, future research should incorporate additional factors that may impact lighting environments. For example, geographical locations, climatic conditions and weather patterns can affect lighting intensity and variability. More accurate and comprehensive analysis of lighting should take these aspects into consideration.

Measures of HSR drivers’ behavior can be more diverse and comprehensive in the future. First, future studies could incorporate both subjective (e.g., questionnaires) and objective (e.g., eye tracking) methods to delve deeper into how HSR drivers’ visual behavior. Insights into road drivers’ behavior have been gathered through both questionnaire surveys and behavioral data, providing a broader and more compelling understanding. Second, future studies could explore the long-term effects of different lighting conditions on HSR drivers’ visual behavior and driving performance, which is critical for HSR operation safety.

## Data Availability

The data presented in this study are available on request from the corresponding author.
